# “Chamber #8” – a holistic approach of high-throughput non-destructive assessment of plant roots

**DOI:** 10.3389/fpls.2023.1269005

**Published:** 2024-01-04

**Authors:** Joelle Claussen, Thomas Wittenberg, Norman Uhlmann, Stefan Gerth

**Affiliations:** ^1^ Fraunhofer Institute for Integrated Circuits (IIS), Department Development Center X-ray Technology, Fuerth, Germany; ^2^ Friedrich-Alexander-Universität Erlangen-Nürnberg, Department for Visual Computing, Erlangen, Germany

**Keywords:** phenotyping, x-ray, CT, non-destructive testing, roots, data management

## Abstract

**Introduction:**

In the past years, it has been observed that the breeding of plants has become more challenging, as the visible difference in phenotypic data is much smaller than decades ago. With the ongoing climate change, it is necessary to breed crops that can cope with shifting climatic conditions. To select good breeding candidates for the future, phenotypic experiments can be conducted under climate-controlled conditions. Above-ground traits can be assessed with different optical sensors, but for the root growth, access to non-destructively measured traits is much more challenging. Even though MRI or CT imaging techniques have been established in the past years, they rely on an adequate infrastructure for the automatic handling of the pots as well as the controlled climate.

**Methods:**

To address both challenges simultaneously, the non-destructive imaging of plant roots combined with a highly automated and standardized mid-throughput approach, we developed a workflow and an integrated scanning facility to study root growth. Our “*chamber #8*” contains a climate chamber, a material flow control, an irrigation system, an X-ray system, a database for automatic data collection, and post-processing. The goals of this approach are to reduce the human interaction with the various components of the facility to a minimum on one hand, and to automate and standardize the complete process from plant care via measurements to root trait calculation on the other. The user receives standardized phenotypic traits and properties that were collected objectively.

**Results:**

The proposed holistic approach allows us to study root growth of plants in a field-like substrate non-destructively over a defined period and to calculate phenotypic traits of root architecture. For different crops, genotypic differences can be observed in response to climatic conditions which have already been applied to a wide variety of root structures, such as potatoes, cassava, or corn.

**Discussion:**

It enables breeders and scientists non-destructive access to root traits. Additionally, due to the non-destructive nature of X-ray computed tomography, the analysis of time series for root growing experiments is possible and enables the observation of kinetic traits. Furthermore, using this automation scheme for simultaneously controlled plant breeding and non-destructive testing reduces the involvement of human resources.

## 1 Introduction and state of the art

In the past years, it has been observed that breeding plants has become more difficult, as the visible difference in phenotypic data is much smaller than decades ago (Costa et al., 2019). With the currently ongoing climate change, it is increasingly necessary to breed new crops that can cope with the shifting climatic conditions (Tester and Langridge, 2010; Field et al., 2014) and will additionally achieve higher yields to feed the growing world population. To find and select good seed candidates for future breeding, whose plants are adapted to the new climate conditions, phenotypic experiments can be conducted under climate-controlled conditions (Langstroff et al., 2022). Based on such phenotypic data, it is possible to obtain important information on the growth, biomass, and yield of a plant under different biotic and abiotic conditions (Shanahan et al., 2001; Golzarian et al., 2011; Tisné et al., 2013; Muraya et al., 2017; Jimenez-Berni et al., 2018). While above-ground traits of plants can be assessed non-destructively with different types of optical sensors, for root growth access to non-destructively measured traits is much more challenging. Even though MRI or CT imaging techniques have already been established for the non-destructive testing of plant roots (Bao et al., 2014; van Dusschoten et al., 2016), these volumetric imaging techniques rely on an adequate infrastructure for the automatic handling of the plant pots as well as the controlled and systematic breeding control. By automated CT-based non-destructive serial observation of below-ground traits, it is possible to quantify the further development of the roots after a particular event, such as, e.g., a biotic or abiotic stress. If such evaluation is done by excavation, only one observation at a certain point of time can be evaluated and information about further developments is lost. When samples are taken, it is also unclear whether that plant part was in the process of growing, stagnating, or dying. With the help of automated non-destructive X-ray technology, it is possible to observe underground plant structures during the growth process. Additionally, objective and quantifiable characteristics for roots such as the root length lroot or the root volume Vroot can be calculated and extracted at a certain observation time. These characteristics can then furthermore be linked to and correlated with other sensor data such as the related climate conditions.

To address these challenges simultaneously – namely non-destructive imaging of plant roots combined with a standardized and highly automated high-throughput approach – a workflow was designed and a novel scanning facility to study belowground root growth was developed. For plant research and development, it is essential to evaluate the root growth. Characteristics related to individual growth progression are of particular interest here.

Our “*Chamber #8*” has a holistic approach with a very high level of automation and will be described in detail in Section 3. On one hand, it consists of dedicated hardware (Section 3.1), including a climate chamber, an irrigation system, and an X-ray system (with source and target) combined with a turntable to obtain multiple projections of the roots. On the other hand, a software system (Section 3.2) maps the workflow and process control of all the plants. This is all integrated into a database to connect and document all information coming from the automatic data collection (including pot RFID and barcodes), the climate conditions, and the post-processing pipeline. Furthermore, in Section 4, some application examples for high-throughput CT scanning of different types of roots will be provided.

One goal of this holistic approach is to reduce unnecessary, expensive, and error-prone human interaction with the various components of the facility to a minimum, to minimize undocumented bias, and to automate and standardize the complete process from plant care via measurements to the derivation of descriptive parameters about the roots. As a result, the user receives standardized phenotypic traits and properties that were collected objectively.

The non-destructive, image-based acquisition, digitization, and evaluation of plant characteristics (e.g., growth, structure, color, and extension of roots and leaves) with different above- and below-ground image sensors is possible today. A tight correlation of this data with the genotypical expression of the observed plants provides informative results needed for the agricultural industry. There are already phenotyping platforms [APPF (https://www.plantphenomics.org.au/) (Langstroff et al., 2022), available in which various sensors such as RGB cameras (Arvidsson et al., 2011), 3D scanners (Biskup et al., 2007; Scholz et al., 2018), multispectral cameras [APPF (https://www.plantphenomics.org.au/) (Ustin and Gamon, 2010), computed tomography (CT) (Bao et al., 2014; Lafond et al., 2015; Rogers et al., 2016; Herrero-Huerta et al., 2021), and magnetic resonance imaging (MRI) (Metzner et al., 2015; van Dusschoten et al., 2016) are integrated.

In the known applications, the sensors, the climatic chamber, and the conveyor belt are usually from different manufacturers and vendors, which may lead to some problems in data merging. There exist recommendations that metadata need to be connected to measurement data (Krajewski et al., 2015; Ćwiek-Kupczyńska et al., 2016; Tardieu et al., 2017; Von Suchodoletz et al., 2021). Even though there already exists some progress in connecting the acquired image and metadata and making them reusable (Coppens et al., 2017; Bolger et al., 2018), there still remain some challenges. Therefore, the complex phenotyping data of the investigated plants are often unavailable due to a lack of infrastructure to store and process the data as well as a known lack of standardized data structures, and data format persistence. Known data semantics and automated approaches for data analysis and data representation are often difficult and error-prone in dealing with the longitudinal aspect of growth volume data. Hence, a close collaboration between data managers, data scientists, and sensors is essential to drive the development of information systems that enable data reusability.

To illustrate the operation of “chamber #8”, we show three examples that are relevant for the community of plant breeders.

## 2 Methods

In this section, all necessary hardware components (Section 3.1) as well as the control software (Section 3.2) are described. Furthermore, it will be shown how all these components interact with the control software.

### 2.1 Hardware components

**Figure 1:**
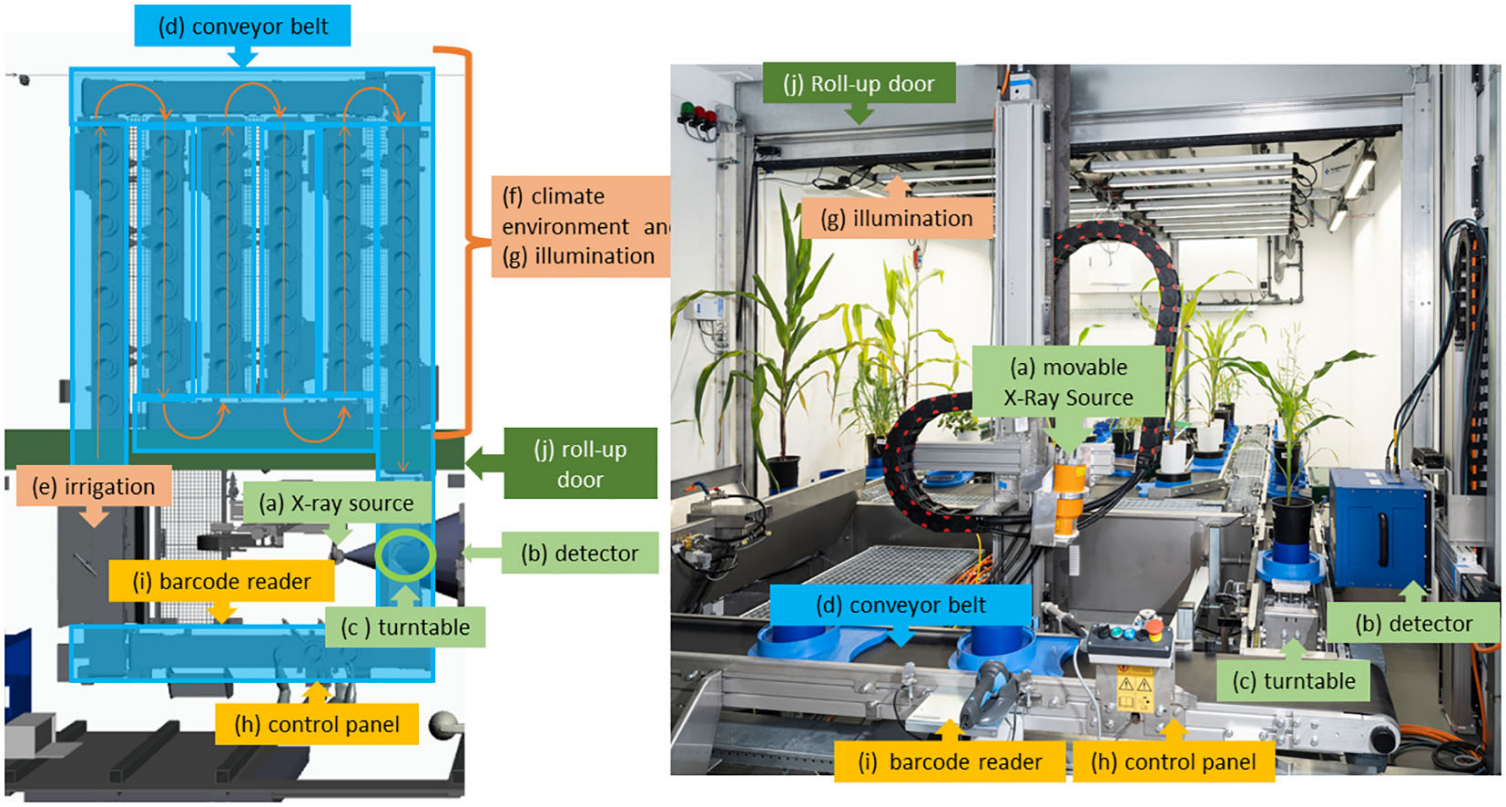
Different views into “chamber #8”- Left side: CAD illustration from top view, right side: view into the chamber. All green components belong to the X-ray system, the yellow and blue to the conveyor belt and the orange ones to the climate camber. The central elements are the (movable) X-ray tube **(A)**, the detector **(B)**, and the turntable **(C)** between them. Separated by a roll-up door **(J)**, in the back side of the climated chamber **(F)** and illumination systems **(G)** are installed. From the climate section **(F)** the plants in their pots are moved by a conveyor belt **(D)** to the turntable **(D)** and back. At the front the control panel **(H)** and the barcode reader **(I)** are located. Also in the front section, the irrigation system **(E)** with sensors is placed.

As depicted in **Figure 1**, in “chamber #8”, a controlled climate environment (f) with an automated conveyor belt (d), irrigation (e) and illumination (indicated in brown) and X-ray equipment (a), (b) is integrated into a radiation protective housing. This offers the advantage that the plants do not have to leave the climate environment for irrigation and CT measurement. On the left side, **Figure 1** shows a CAD file view of “chamber #8” from above while on the right side a view of “chamber #8” is depicted through the open radiation shield door. The controlled climate environment (f) with illumination (g) is installed in the back of the room. The capacity of the conveyor belt (d) is 42 plants. The front part of “chamber #8” is separated from the climate section by a roll-up door (j) and houses the irrigation system (e) and the X-ray system (a), (b), (c). The roll-up door (j) is used to protect the sensors in high humidity conditions when measurements are not being taken. **Figure 1** depicts the individual main technical components used in “chamber #8”, specifically (a) the X-ray tube with lifting axis, (b) the detector with lifting axis and horizontal axis, (c) the turntable integrated into the conveyor belt, (d) the plants on the conveyor belt, (e) the irrigation system, (f) the climate environment and (g) illumination, (h) the control panel for loading the conveyor belt and (i) the barcode reader. The overall system design and integration were done in close cooperation with the company Phenokey. The climate chamber was manufactured by Bosman Van Zaal (The Netherlands) and the conveyor belt by Flier (The Netherlands). Within the climate section of “chamber #8” it is possible to set and control values for temperature, lighting conditions, CO_2_ levels, and air moisture. The preset values of the climate conditions are available at “LetsGrow.com” (The Netherlands) and are connected to the CT measurements of the plant.

The engineering of the X-ray system has been designed to perform fast CT measurements. Therefore, a high-power X-ray source (“Comet 225 HP/11”, see **Figure 1A**) is used. This high-power source is used to enable fast measurements and illuminate the detector within a very short timeframe. The detector (“Xeye2530”, by Fraunhofer EZRT, **Figure 1B**) (Scholz et al., 2013; Nachtrab et al., 2014) was developed in such a way that it has a long service life despite continuous operation. This is achieved via shielding all electrical components from direct or scattered X-ray radiation. Thus, the detector shows no or only a very low degradation. The size of the detector is designed to scan plant pots with a diameter of up to 20 cm and a height of 20 cm within a single measurement. The native pixel pitch of the detector is 45 µm and the scintillator material is CsI. To increase the scanning speed further, the detector is binned to a pixel pitch of 90 µm. With this configuration, the lowest achievable voxel edge length is 75 µm voxel edge length for the reconstructed dataset. This voxel edge length is derived from the binned pixel pitch divided by the magnification. In the binned configuration, the detector has 3328x2777 pixels in total.

The turntable (**Figure 1C**) was designed to achieve the needed precision of rotation and to fit into the conveyor belt. The turntable has an angular accuracy of 400 µrad, with a repetition accuracy at the rim of the table of 60 µm, a maximum diameter of 150 mm to fit in the conveyor belt, and a maximum axial load capacity of up to 10 kg.

Prior to the scan, the pot carrier moved over the turntable and stopped at the measurement position. To place the plant onto the adapter plate on the turntable, both the belt and the stopper are lowered. As each carrier has a fin on the backside, it was a challenge to place the plant as close as possible to the detector, but at the same time not to get the fin captured by the detector. Additionally, we wanted to specifically capture the bottom of the pot within the reconstructed 3D volume. This was achieved by accurately placing the carriers on the turntable and elevating the plant inside the carrier.

Both the X-ray source (**Figure 1A**) and detector (**Figure 1B**) are mounted on linear axes to be flexible in height. This provides the possibility to scan elongated objects with a maximum height of up to 160 cm if several measurements are stacked vertically. The integrated use of the turntable allows a complete, synchronized, and automated 360 degrees or limited angular scan of the plants’ roots in the pots, including the bottom of the pot, where – after some time – a lot of roots are visible. Additionally, the detector (**Figure 1B**) is mounted on a horizontal linear axis to provide a larger field of view (FoV), if a scanning procedure with detector displacement is needed. The turntable and the linear axes of the X-ray source and detector are controlled by a Programmable Logic Controller (PLC) to ensure security.

### 2.2 System control software

**Figure 2:**
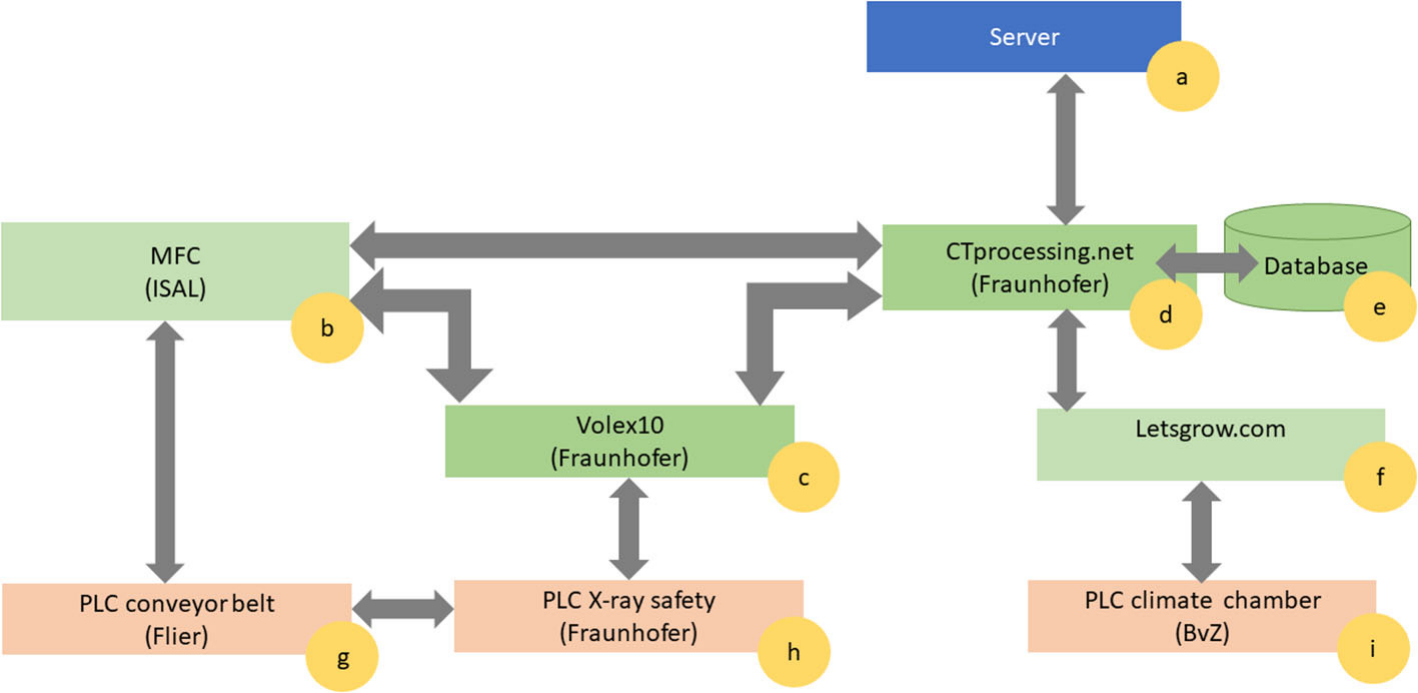
Communication flow between the different Programmable Logic Controllers (PLCs) marked in orange, Software components marked in green, and PC-Hardware marked in blue. There is **(A)** the server, where all the data are processed. The central elements of the software ecosystem are the Material Flow Control ISAL **(B)**, the X-ray control software Volex 10 **(C)** and the data management CTprocessing.net **(D)**. Additionally, there is a database storing all the processing tasks information and system metadata **(E)** connected with the climate database **(F)**. The PLC layer controls the conveyor belt **(G)**, the X-ray safety **(H)** and the climate chamber **(I)**.

**Figure 2** illustrates the communication between the different PLC units (**Figures 2G–I**) and the software components (**Figures 2B–D, F**). All acquired information about the plants and the sensors, and the scan is collected and stored in a joint database (part of the “CTprocessing.net” module, **Figure 2E**) in order to have all necessary data persistent and available for the user.

The material flow controller (MFC) (**Figure 2B**) consists of the software ISAL (Indigo Logistics). ISAL provides the interface to the conveyor belt PLC (**Figure 2G**). Each carrier in the conveyor belt has a unique RFID tag and relates to a barcode placed on the plant pot. At any point in time, the ISAL software is able to display which plant – respectively pot – is currently at which location within “chamber #8” and which events (irrigation, scanning) have been carried out recently or are planned next. For automation purposes, the planning of the measurements and the irrigation is created within the ISAL framework (**Figure 2B**). To this end, individual planning can be done per plant or individual plants can be grouped for a certain experiment. For the irrigation of the plant, the timing and duration can be specified as parameters.

The planning for the X-ray measurements can also be done using ISAL (**Figure 2B**), which communicates with the X-ray control software *Volex10* (Fraunhofer IIS) (**Figure 2C**) (Fíla et al., 2015; Kumpova et al., 2015). *Volex10* also communicates with the individual components of the X-ray system, the X-ray safety PLC (**Figure 2H**) as well as the post-processing software (“CTprocessing.net”, **Figure 2D**) to collect and store all captured data. Furthermore, to ensure safe operation at all times, the conveyor belt PLC (**Figure 2G**) is connected to the X-ray safety PLC (**Figure 2H**). Thus, it is possible to disable interlock and safety circuits simultaneously according to national and international CE regulations.

With this integration on the hard- and software level, the parameters for the CT measurement can be selected directly within ISAL (**Figure 2B**). In advance, necessary CT parameters are stored within the *Volex10* software (**Figure 2C**). CT parameters include tube parameters (voltage *V*, current *C*, prefilter), detector parameters (exposure time *E*), and procedure-specific parameters (number of projections, speed, and the positions of the various axes). *Volex10* has been developed to be used for automated as well as manual measurements (Fíla et al., 2015; Kumpova et al., 2015). This provides the user with the flexibility to make individual measurements of other objects between the automated measurement intervals controlled by the ISAL software (**Figure 2B**).

When a CT measurement has been made, the *Volex10* software (**Figure 2C**) writes all related parameters as well as the captured X-ray projections (raw data) into the database (**Figure 2E**). Afterward, the “CTprocessing.net” software (Fraunhofer EZRT) (**Figure 2D**) takes care of the automated processing and 3D reconstruction of the acquired X-ray image data. This processing includes artifact corrections in the raw data, reconstruction to a 3D volume as well as the segmentation of the 3D volumes. The selection and parameterization of the individual processing steps can be customized. Currently, the segmentation of roots (Gerth et al., 2021; Alle et al., 2023) and the segmentation of “grain ears” (Schmidt et al., 2020) are supported by the automated workflow, although other algorithms can easily be inserted into the concept with an easy Python API.

For the root segmentation with RootForce the following traits can be calculated, which are explained in Gerth et al., 2021 in more detail (Gerth et al., 2021): Total root volume V_root_ (mm^3^), Total root length L_root_ (mm), 25% Rootmass quantile depth (mm), 50% Rootmass quantile depth (mm), 75% Rootmass quantile depth (mm), 90% Rootmass quantile depth (mm), Form fraction F (in %), Minimum root angle θ_min_ (in °), Maximum root angle θ_max_ (in °), Mean root angle θ_mean_ (in °), Mean root density (gray intensity values), Mean soil density (gray intensity values), and soil/roots density relation factor (in %). The software “CTprocessing.net” (**Figure 2D**) has been developed to provide automated post-processing possibilities as well as to monitor the current state of the X-ray projection data. It is flexible and scalable. In the background, a database (**Figure 2E**) is running, in which the X-ray projection data sets and the corresponding information about the individual processing steps are stored for quality assurance and backtracking. Clients running on different servers (“workers” **Figure 2A**) within the network, process the individual steps as jobs. The network of computers can quickly be expanded to include additional “workers”. Once a certain job has finished the database is automatically updated. On a dashboard, the user receives a status overview of the data and jobs.

The automated data post-processing step is motivated by the huge amount of captured raw data. If, e.g., an experiment is planned over six weeks with three measurements per week and a maximum of 42 plants to be scanned, these scans sum up to a total of 756 raw data sets with a data size of 16 Terabyte each. This yields another 14 Terabyte for the reconstructed volumes with a resolution of 76 µm.

The post-processing pipeline within the “CTprocessing.net” software (**Figure 2D**) is designed to be flexible. The basic post-processing components are the 3D-reconstruction and the segmentation of the 3D data. Depending on the experiment at hand, the image processing pipeline can be configured individually. For example, correction possibilities for ring artifacts, beam-hardening, or other artifacts can be computed before the 3D reconstruction is made. Additionally, after the traits of the roots are calculated, a visualization of the traits can be added to the pipeline.

Within the “CTprocessing.net” software (**Figure 2D**) the actual climate conditions are stored as metadata and are connected to the individual plant. There exists a programming interface to the above-mentioned “Let’s grow” software to import the plants’ data such as temperature, light conditions, CO2 level, and air moisture. Additionally, the irrigation time points are also stored as metadata within the “CTprocessing.net” database. This plant-oriented data collection enables the user to have a holistic understanding of the environment during the measurement and use this knowledge for the interpretation of the calculated traits of the CT-measurements.

## 3 Results

In the following part, we show three examples of experiments that can be done with “chamber#8”. The exact experimental details are shown in separate publications.

### 3.1 Example 1: longitudinal potato growth

In the work by *van Harssellar et al.* (Van Harsselaar et al., 2021) it was shown that, e.g., the growth of potatoes can be studied under stress conditions using CT imaging. Within this study, the tuber growth was analyzed, and the acquired CT data was linked with the sampled data such as qRT-PCR and SuSy-activity. In two experiments, five different potato genotypes (“*Agria*”, “*Saturna*”, “*Tomensa*”, “*Ramses*”, and “*Diamant*”) with four replicates were scanned every second day in the CT system of “*chamber #8*” over a period of seven weeks and the collected data was evaluated semi-automatically. The described hardware and software automation approach within “*chamber #8*” has made it possible to observe that individual tubers start growing again after drought and heat stress. The volume and the density were calculated out of the CT data, therefore the virtual biomass could be derived. The calibration to dry weight is conducted with sampled reference data. The exact calculation is explained in the work by *van Harssellar et al.* (Van Harsselaar et al., 2021).

**Figure 3:**
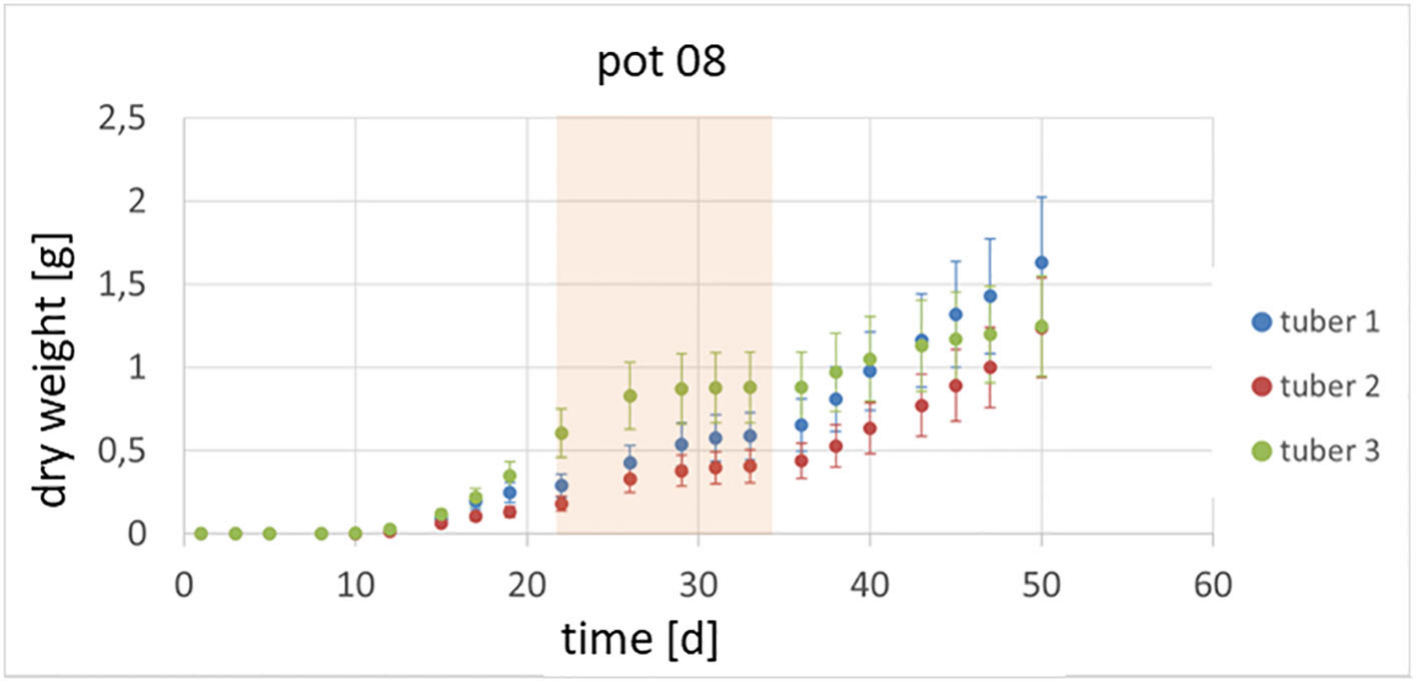
Growth of tubers within one plant is shown. Between day 22 and day 35 the plants were exposed to a combined heat and drought stress (marked in orange).

**Figure 4:**
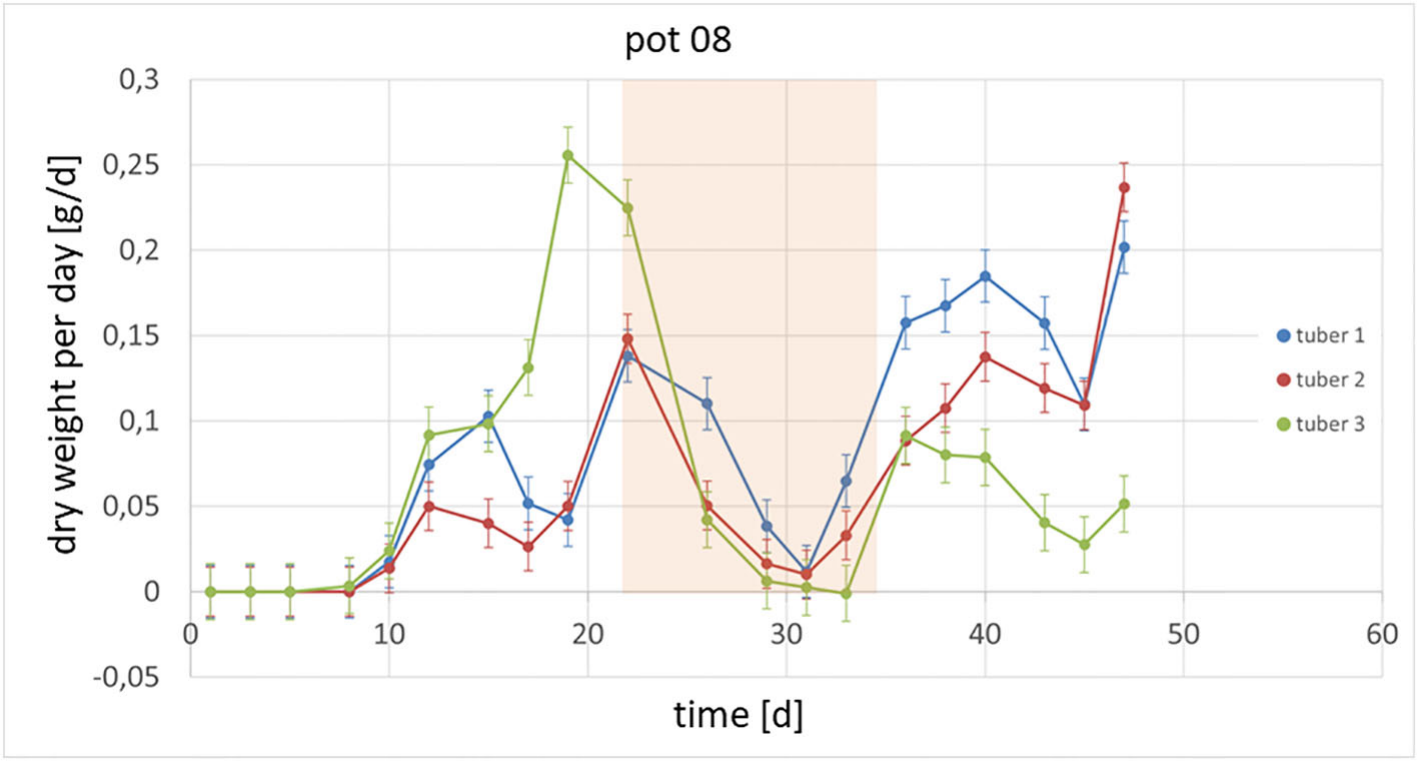
Growth per day of tubers within one plant is shown. Between day 22 and day 35, the plants were exposed to a combined heat and drought stress (marked in orange).

It could also be shown that the investigated genotypes show different strategies in which tubers continue to grow or whether there are victim tubers. In **Figure 3**, an example of the growth of potato plant tubers is depicted. Between day 22 and day 35, the observed plant was exposed to heat and drought stress and hence the growth of the tubers was slowed down or even stopped. Starting at day 36, the tubers resumed growth at different rates due to the fact, that the climate was changed back to previous conditions. In **Figure 4** the growth rate in dry weight per day is displayed for the same pot as above.

### 3.2 Example 2: storage root development of cassava plants

**Figure 5:**
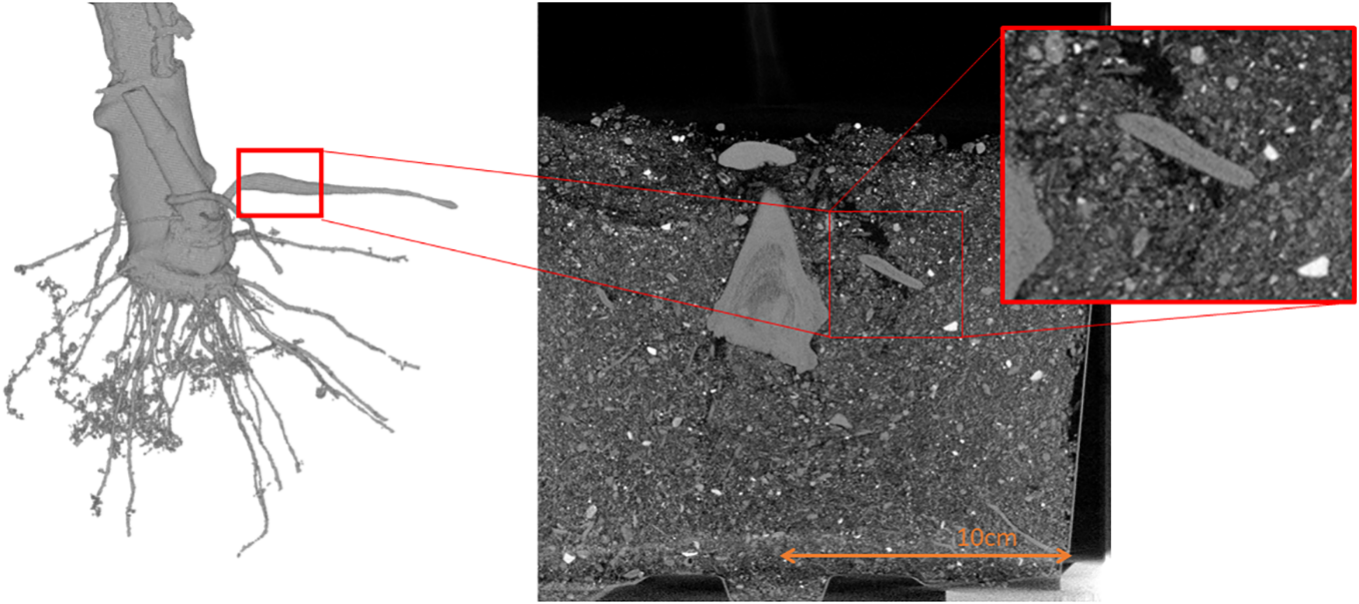
Shows the density drop within a storage root. On the left side a 3D image is depicted and on the right side a cross section of the 3D measurement.

Experiments with *cassava plants* (grown from stem planting) were made to investigate if there exists a criterion to distinguish potential storage roots. The results have shown that through the acquisition of time series the emergence of storage roots can be observed (Carluccio et al., 2022) while it can be seen that fiber roots remain constant. The emergence of the low-density channel was observed, and it could also be shown that in the storage roots, this low-density channel always emerges. Even if the bulking has not started yet, potential storage roots already depict a low-density channel (see **Figure 5**) in contrast to fiber roots, which do not have these structural features. These observations match with the reference experiments, being a destructive analysis of storage roots, but could not yet be proven due to the limited number of replicates. Hence, in this experiment, the non-destructive nature of CT measurements was used to observe the growth dynamics and structural changes during the bulking process, by the evaluation of the acquired metadata of the plants stored within the “CTprocessing.net”- database.

### 3.3 Example 3: different genotypes of maize plants

**Figure 6:**
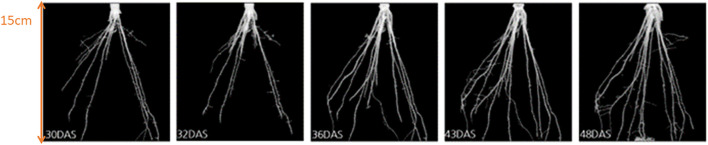
Growth of maize roots (of genotype A3) under controlled conditions. From left to right: segmented roots at 30, 32, 36, 43, and 48 days after sowing (DAS).

**Figure 7:**
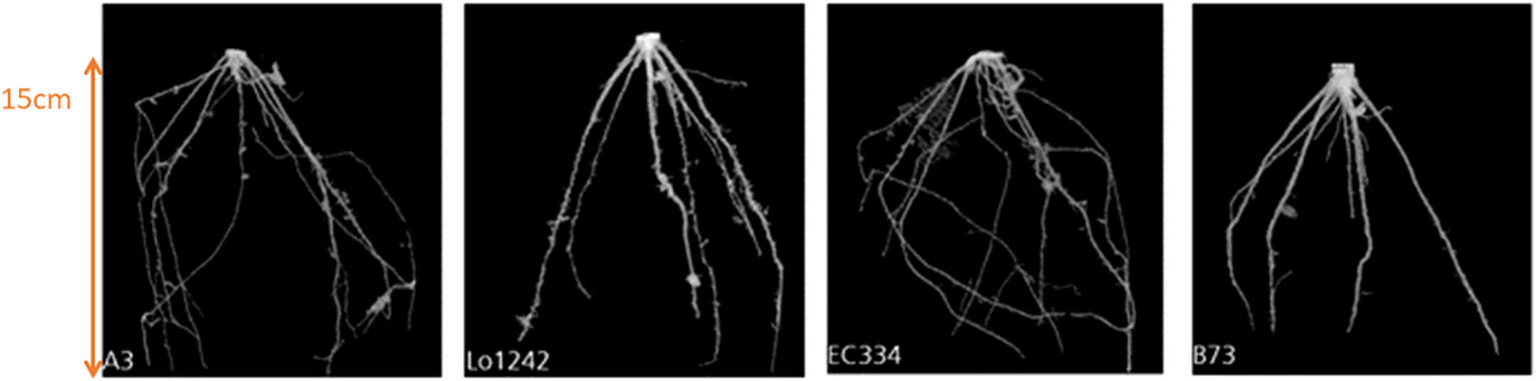
The spatial root development of four different genotypes (A3, Lo1242, EC334, B73) at the same observation time (36 DAS) under combined nitrogen and water stress.

In comparison to previously conducted manual experiments, using the automation system installed in “chamber #8”, we can now scan and measure up to 42 plants within 8 hours (compared to 20 hours using manual labor). Furthermore, the analysis of various root systems such as *maize roots* is now possible using the automated post-processing step for 3D root segmentation (Gerth et al., 2021; Alle et al., 2023). For the analysis of *maize roots* 16 genotypes (*A554, B104, Lo1261, IDT, Oh02, W23, Lo1106, Lo1282, A554, B104, Lo1261, B73, Oh02, W23, Lo1106, Lo1282*) were used with five replicates each under combined nitrogen-water stress conditions. In **Figure 6** an example of the growth of a time series of genotype A3 under control conditions is shown for 30 DAS (days after sowing) until 48 DAS. **Figure 7** depicts an example of four genotypes (*A3, Lo1242, EC334, B73*) at the same time point (36 DAS) under combined nitrogen and water stress. This example shows that within the 3D segmentation step not only big belowground organs like storage roots or tubers can be segmented automatically, but also root systems such as maize. It has to be noted, that within this experiment a pot diameter of 17cm was used, and therefore finer lateral roots are not automatically detectable within the automated “chamber #8”. Depending on the pot diameter and the soil used, lateral roots are detectable. However, the diameter of the lateral root system of maize is on the resolution limit “chamber #8” is capable.

**Figure 8:**
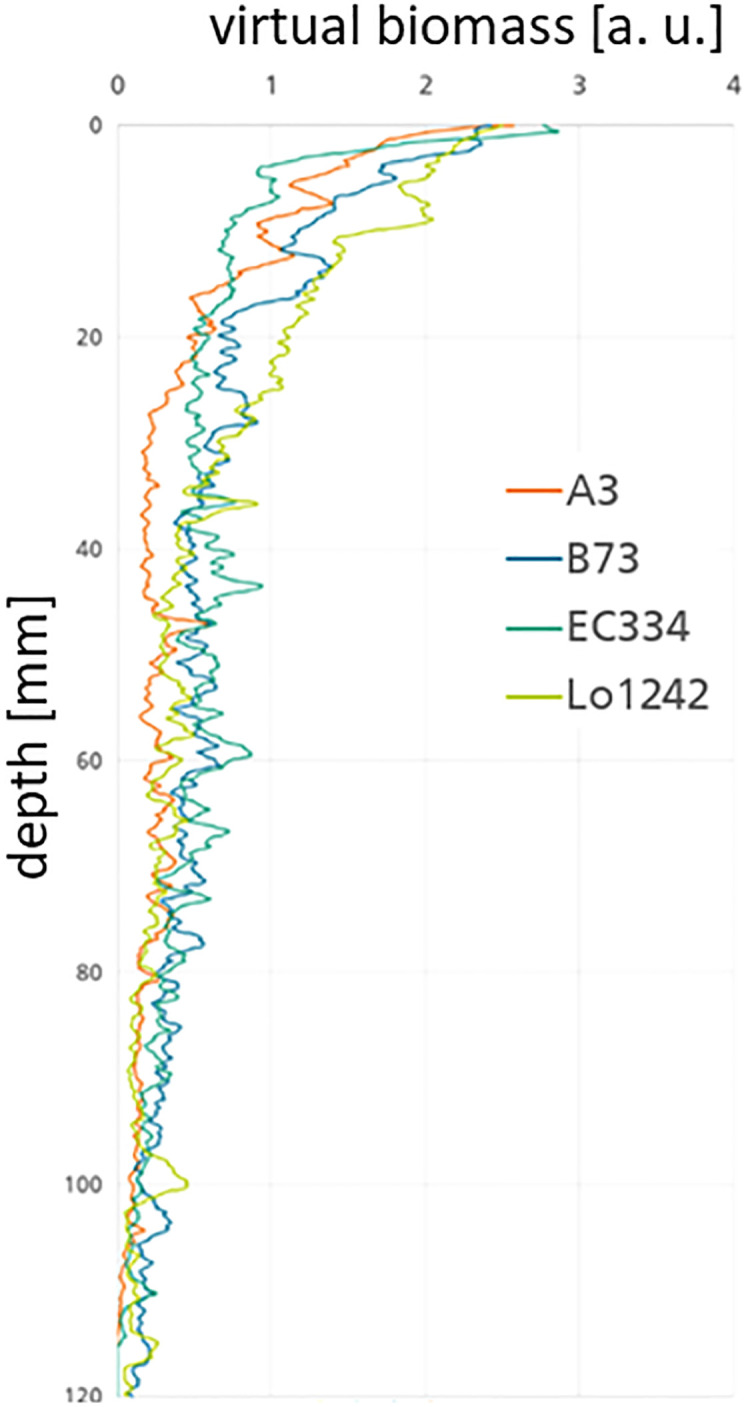
Relation of computed biomass B over root depth (lroot) in the pot for four different genotypes (A3, Lo1242, EC334, and B73) under combined nitrogen and water stress. Lo1242 has more biomass in the upper part of the pot than the other genotypes.

With the collected data stored in the database (**Figure 2E**) it is also possible to plot and visualize the traits calculated from the post-processing pipeline. Typical and helpful values to be plotted are, e.g., the virtual *biomass B vs*. the *root depth d*_root_ (see **Figure 8**). This virtual biomass is in most cases in arbitrary units [a. u.] due to the fact that the individual grey values are not normalized by a phantom and therefore do not represent a direct physical measure. Using the grey values and the segmented root volume a number tightly correlated with the weight can be calculated from the CT measurements. In contrast to the most often used root length density gathered from segmented 2D images, we could in principle calculate a root volume density map. For this, you would need to take into account the volume of the pot to receive the root volume density at each depth. However, having the benefit of receiving an attenuation coefficient tightly correlated with the physical density within a root, we can directly calculate the virtual biomass as a function of the pot depth by multiplying the attenuation within the root with the total root volume at each depth.

Based on the results of these three examples – longitudinal potato growth (Section 4.1), storage root development of *Cassava* plants (Section 4.2), and different genotypes of maize plants (Section 4.3) – of long-term plant breeding, automated CT scanning and 3D image data segmentation experiments, it becomes clear that – *without* any automation – the personnel resources for all the needed measurements (as well as for plant transport, watering, …) are extremely high. Furthermore, using manual labor the plants may be involuntarily exposed to varying climatic conditions when they change locations. The described new level of automation within “chamber #8” (Section 3) allows us to study root growth of plants in field-like substrate non-destructively over a defined period and furthermore calculate phenotypic traits of root architecture without user interaction. In total. this automation scheme leads to a higher throughput, fewer errors, and a reduced workload for the involved personnel.

## 4 Discussion

The described and exemplified holistic approach of automated breeding and non-destructive 3D scanning enables the user to use X-ray sensors in a high-throughput and completely automated manner. The goal for the development of “chamber #8” was to perform more measurements within a shorter time span with reduced errors and hence to reduce the amount of human-machine interaction as well as human-plant interactions to a minimum. Using this complete automation approach within “chamber #8” the throughput within an eight-hour time slot has been more than doubled compared to a completely manually labored and controlled system. Due to the described automation, it is now possible to measure roots in a “24/7” manner, which increases the throughput even more. The amount of sensor and image data automatically and continuously generated in “chamber #8” is only manageable because the “CTprocessing.net”-software monitors all the image acquisition and image processing steps and distributes the necessary jobs to different “working” computers.

The goal to reduce user interaction (human-machine interaction and human-plant interaction) has two reasons. On one hand the proposed system within “chamber #8” can now be operated without a person on site. This sets the staff free for other tasks, reduces costs, and simultaneously increases the amount of measurements. On the other hand, regular human-made errors – such as misplaced or exchanged pots, wrong naming of data, or falsely set parameters for post-processing – are strongly reduced. Due to the proposed complete automation from breeding to the computation of traits, the error and influence due to wrong parameters is close to zero. As the barcode on the pots is connected to the RFID, the MFC knows the position of the pot, respectively plant, within the climate chamber as well as the watering and X-ray measurement conditions at any time.

## 5 Conclusion

Using the proposed automation scheme within “chamber #8” of controlled plant breeding in combination with automated 3D scanning and image post-processing to characterize the root growth, it has become possible to reduce the number of human-plant interactions as well as the human-machine interactions to a minimum. Hence, possible human-based errors, such as data labeling, or process parametrization have been eliminated and furthermore, the time needed for the scanning process could be reduced from over twenty hours to eight hours.

In our experiments, we have observed and characterized the longitudinal root growth of cassava, potato, and maize plants. Nevertheless, other types of plants such as soybeans or wheat can also be investigated.

To extend the measuring possibilities, it is planned to integrate additional sensing devices like 3D-, hyperspectral, or near-infrared (NIR) sensors into “chamber #8” in the near future. These sensors will provide extended phenotypic data of the above-ground traits of the investigated plants. Nevertheless, all these additional above-ground sensors have to be integrated and synchronized within the described hardware and software processing pipeline. This extended data collection will yield an even more holistic view of the plants. Additionally, automated below- and above-ground sampling with a robot (Alenya et al., 2011; Foix et al., 2015; Alenya et al., 2013; Bao et al., 2017; Bao et al., 2018) could possibly be integrated in a second step into “*chamber #8*” to further reduce the human workload.

High-throughput phenotyping platforms produce substantial amounts of data that are very valuable for plant breeding and can be used in statistical prediction models. Even though there is substantial progress in the connection and reusability of data there are still many challenges and opportunities ahead.

## Data availability statement

The data analyzed in this study is subject to the following licenses/restrictions: The size of the datasets for these experiments is in total 150 TB. Therefore, they are only available on request to the corresponding author. Requests to access these datasets should be directed to Joelle Claussen, joelle.claussen@iis.fraunhofer.de.

## Author contributions

JC: Conceptualization, Data curation, Formal analysis, Investigation, Project administration, Visualization, Writing – original draft. TW: Conceptualization, Supervision, Visualization, Writing – original draft, Writing – review & editing. NU: Funding acquisition, Supervision, Writing – review & editing. SG: Conceptualization, Funding acquisition, Methodology, Supervision, Writing – review & editing, Resources.
